# Molecular and serological characterization of hepatitis B vaccine breakthrough infections in serial samples from two plasma donors

**DOI:** 10.1186/s12985-019-1154-4

**Published:** 2019-04-03

**Authors:** Mary C. Kuhns, Anne L. McNamara, Vera Holzmayer, Gavin A. Cloherty

**Affiliations:** Infectious Disease Research, Diagnostics Division, Abbott Laboratories, Dept. 09NC, Bldg. AP20, 100 Abbott Park Road, Abbott Park, IL 60064 USA

**Keywords:** Hepatitis B virus, Hepatitis B surface antigen, Vaccine breakthrough, Hepatitis B surface antigen mutations

## Abstract

**Background:**

Although vaccines for hepatitis B virus (HBV) are highly effective, HBV infections in vaccinees occur. Index samples of breakthrough infections are typically anti-HBc negative but HBV DNA positive with protective anti-HBs levels while HBsAg detection may be delayed or absent. HBsAg mutations have been associated with some vaccine breakthrough cases.

**Methods:**

This research characterizes the serological and molecular profiles of vaccine breakthrough infections in serial samples from two commercially available plasma donor panels. Samples were tested with commercially available assays for HBV antigens and antibodies: HBsAg, HBeAg, anti-HBc, anti-HBc IgM, anti-HBe, and anti-HBs. Different immunoassay approaches for earlier detection of breakthrough infection were explored including hepatitis B core-related antigen (HBcrAg), a research assay for preS2 antigen, and a new prototype ARCHITECT HBsAg assay with improved sensitivity. The prototype HBsAg assay is fully automated and involves no sample pre-treatment. Molecular testing included HBV DNA quantitation and sequencing of preS1, preS2, surface, and basal core promoter/core promoter genes.

**Results:**

Although the research preS2 antigen assay allowed earlier detection of the breakthrough infections than current HBsAg assays and HBcrAg, the new prototype ARCHITECT HBsAg assay provided the earliest serologic detection. The ability of the new prototype HBsAg assay to detect HBsAg in the presence of anti-HBs was investigated using known concentrations of native HBsAg mixed with anti-HBs from a vaccinee. The results demonstrated that the prototype ARCHITECT assay is more sensitive in detecting HBsAg in the presence of anti-HBs than current HBsAg assays. Sequencing revealed multiple substitutions in preS1, preS2, and S regions for one panel including a rare D144N substitution associated with vaccine breakthrough that emerged with increasing frequency as the breakthrough infection developed.

**Conclusions:**

When compared with other immunoassay approaches, the new prototype ARCHITECT HBsAg assay allows earlier detection of vaccine breakthrough infections and more sensitive detection of HBsAg in the presence of anti-HBs. Molecular characterization of longitudinal samples demonstrated the progressive appearance of a rare HBsAg mutation associated with vaccine breakthrough.

## Background

Vaccines for hepatitis B have been available since the 1980’s beginning with the first plasma derived vaccine and progressing to vaccines utilizing recombinant hepatitis B surface antigen (HBsAg). Over 90% of adults respond to a full vaccine course with protective levels of antibody to HBsAg (anti-HBs) greater than or equal to 10 mIU/ml [1, 2]. Although the vaccines are highly effective, hepatitis B virus (HBV) infections in vaccinated individuals have been described [3–9].

Vaccine breakthrough infections differ from typical acute HBV infection where the order of appearance of viral markers in the peripheral blood follows a consistent pattern with hepatitis B viral DNA (HBV DNA) followed by HBsAg, hepatitis B e antigen (HBeAg), and antibody to hepatitis B core antigen (anti-HBc). Resolution of infection in typical acute infection is marked by loss of serum HBsAg and HBV DNA and the appearance of anti-HBs. In contrast, during breakthrough infections, HBV DNA becomes detectable in vaccinated individuals who have protective levels of anti-HBs. HBsAg detection may be delayed, transient, or absent.

Two commercially available plasma donor seroconversion series were previously identified as probable breakthrough infections in a study of immune responses to HBV infection in vaccinated and unvaccinated blood and plasmapheresis donors [10]. Breakthrough infection is defined as the presence of HBV DNA and protective levels of anti-HBs in the absence of anti-HBc in the index donation. The aim of the present study was to expand the studies on these longitudinal panels including extensive molecular and serological characterization. In addition, we investigated the use of other HBV immunoassays for early detection of vaccine breakthrough including hepatitis B core-related antigen (HBcrAg), HBV preS-2 antigen, and a new prototype ARCHITECT HBsAg assay. Lastly, we compared the new prototype assay with current HBsAg assays for detection of HBsAg in the presence of anti-HBs.

## Materials and methods

### Study samples

Two commercially available, longitudinally collected acute HBV infection panels purchased from Zeptometrix Corporation (Franklin, MA) were identified as vaccine breakthrough cases as previously described [10]. The index donations of these plasma donor seroconversion panels (Zeptometrix Panels 6272 and 11,000) were positive for HBV DNA and anti-HBs but negative for HBsAg and anti-HBc. The assays used in the initial screening were PRISM HBsAg, PRISM HBcore, Corzyme, AxSYM AUSAB, and Abbott RealTime HBV [10].

To prepare samples containing both HBsAg and anti-HBs, a native HBsAg positive sample (purchased from Serologicals Corp., Atlanta, GA) and a high titer human anti-HBs positive sample from a vaccinee (purchased from North American Biologicals Inc., Boca Raton, FL) were diluted in normal human plasma and combined to achieve HBsAg concentrations of 0.06, 0.11, and 0.23 IU/ml with concomitant anti-HBs concentrations ranging from 0 to 10,000 mIU/ml. The combined antigen and antibody samples were stored overnight at 4^o^ C and then frozen at -20^o^ C.

### Serologic and molecular testing

Samples were tested for HBsAg using ARCHITECT HBsAg Qualitative II (analytical sensitivity 0.017–0.022 IU/ml, Abbott, Sligo Ireland), ARCHITECT HBsAg (quantitative assay, sensitivity 0.05 IU/ml, Abbott, Sligo, Ireland), Elecsys HBsAg II (sensitivity 0.033 IU/ml, Roche, Mannheim, Germany), ADVIA Centaur HBsAg II (analytical sensitivity 0.018 IU/ml, Siemens Healthcare, Erlangen, Germany), and LIAISON XL MUREX HBsAg Quant (analytical sensitivity 0.05 IU/ml, DiaSorin, Saluggia, Italy). Assay sensitivities are those reported in the respective package inserts. All testing was performed as recommended in the respective assay package inserts. In addition, samples were tested with a new prototype ARCHITECT qualitative HBsAg assay with improved analytical sensitivity of 0.0052 IU/ml, improved mutant and genotype detection, and specificity of 99.97–100% [11]. The assay is fully automated and requires no sample pretreatment.

The plasma donor seroconversion panels were tested with an ARCHITECT research assay for pre-S2 surface antigen using two monoclonal antibodies for capture and detection directed at different regions of the preS2 antigen. Anti-HBc, anti-HBc IgM, HBeAg, antibody to hepatitis B e antigen (anti-HBe), and quantitative anti-HBs were evaluated using commercially available ARCHITECT assays (Abbott, Sligo Ireland or Wiesbaden, Germany). HBcrAg testing was performed with the Lumipulse G HBcrAg assay (Fujirebio, Tokyo, Japan). Quantitative HBV DNA levels were determined with Abbott RealTime HBV (Abbott Molecular, Des Plaines, IL).

### Sequencing of the PreS1-PreS2-S gene region

HBV DNA was extracted from 0.5 mL of plasma using an automated DNA sample preparation protocol DNA-protK-500-50 (research use only) on the *m*2000*sp* system (Abbott Molecular, Des Plaines, IL). First- and second-round PCR were performed to amplify the preS1-S region using Amplitaq Gold® DNA polymerase (Applied Biosystems, Foster City, CA). The first-round primers were HBV2813F (5′- TCATTTTGTGGGTCACCATATT-3′, nt 2811–2832) and 18R (5′-CCCATGAAGTTTAGGGAATAAC-3′, nt 860–881); the second-round primers were HBV-2822F (5′- GGGTCACCATATTCTTGGGAAC-3′, nt 2820–2841) and 19R (5′- GTTAGGGTTTAAATGTATACCC-3′, nt 822–843) amplifying a 1245 base pair fragment. The 50 μl PCR reaction contained 0.4 μM of each primer, 2.5 mM MgCl_2,_ 0.8 μM dNTP mix and 25 μl extracted DNA for the first-round PCR, or 2 μl of the first-round PCR as a template for the second-round. First- and second-round amplifications consisted of preincubation at 95 °C for 10 min, 40 cycles of 95 °C for 20 s, annealing at 50 °C for 45 s, extension at 66 °C for 2.5 min for the first-round or 1.5 min for the second round, and final extension at 72 °C for 10 min. Amplified products were purified with ExoSAP-IT® (Affymetrix, Cleveland, OH) and both strands were sequenced directly using the BigDye® Terminator v3.1 Cycle Sequencing kit and the ABI 3130*xl* Genetic Analyzer (Applied Biosystems, Foster City, CA). Sequence data were assembled and edited using Sequencher software (version 5.4.6; Gene Codes Corporation, Ann Arbor, MI). Positions with sequence ambiguities were assigned the appropriate IUPAC designation. Genotype was determined by phylogenetic analysis using the PHYLIP v3.5c software package (J. Felsenstein, University of Washington, Seattle, Washington). Nucleotide sequences were aligned with the reference sequences representing genotypes A-I using BioEdit 7.0.4.1 [12].

### Mutation analysis

Atypical amino acid substitutions in the preS1, preS2, and S genes were determined by comparing the specimen sequences to the genotype A consensus sequence created in BioEdit from the alignment of 1335 genotype A sequences downloaded from https://hbvdb.ibcp.fr [13]. In case of a mixture of wild type and atypical amino acids, a percentage substitution was estimated proportional to the heights of corresponding chromatograms in a mixed nucleotide call. Molecular analysis for precore, basal core promoter, and drug resistance mutations was performed at a commercial reference laboratory by PCR amplification and sequencing of the respective regions.

## Results

### Molecular and serological profiles for panels 6272 and 11,000

**Fig. 1:**
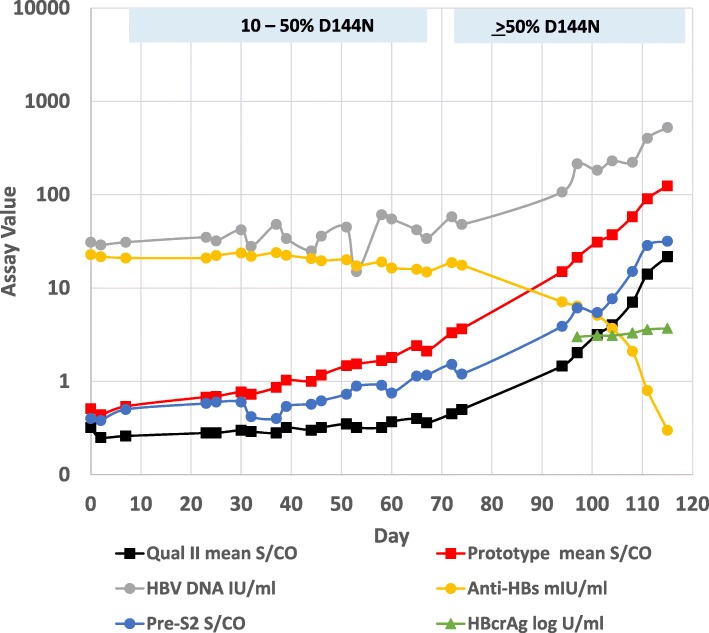
Serological and molecular profiles for panel 6272. Panel 6272 was tested with ARCHITECT HBsAg Qual II, a prototype ARCHITECT HBsAg assay, a research assay for preS2 antigen, ARCHITECT Anti-HBs, Lumipulse HBcrAg, and Abbott RealTime HBV. An S/CO value ≥ 1.0 was considered reactive for the HBsAg and preS2 assays. The limit of quantitation of the HBcrAg assay is 3 log U/ml; results for samples prior to day 97 were below the measuring range of the assay and were omitted from the figure. Anti-HBs levels ≥ 10 mIU/ml are considered protective. The bars above the 6272 graph show the percentage of codons with the HBsAg D144N mutation

Fig. 1 shows the progression of molecular and serological markers for plasma donor 6272 (genotype A1). The first panel member was positive for HBV DNA (31 IU/ml) and anti-HBs (22.8 mIU/ml) but negative for HBsAg by ARCHITECT HBsAg Qualitative II. HBV DNA levels were low and fluctuating for the first 74 days and then began a steady increase coincidental with a marked decline in detectable anti-HBs. Total anti-HBc, anti-HBc IgM, HBeAg, and anti-HBe remained negative throughout the 115 days that this donor was followed. The presence of HBV was first detected by commercially available HBsAg assays at day 94 or 97, depending on the assay used (Table 1).

**Table 1:** Detection of Panels 6272 and 11,000 by Serological Assays

Assay	First Positive Panel Member			
	Panel 6272		Panel 11,000	
	Panel Member	Day	Panel Member	Day
ARCHITECT HBsAg Prototype	12	51	1	0
PreS2 Research Assay	16	65	4	14
ARCHITECT HBsAg Qual. II	20	94	6	21
Elecsys HBsAg II	20	94	6	21
LIAISON XL MUREX HBsAg Quant.	20^a^	94	6^b^	21
Lumipulse HBcrAg	21^c^	97	6^d^	21
Centaur HBsAg II	21	97	6	21

The ARCHITECT Prototype, PreS2, ARCHITECT Qual.II, Elecsys, and Centaur assays are qualitative; the LIAISON and Lumipulse assays are quantitative

^a^0.08 IU/ml HBsAg, ^b^0.05 IU/ml HBsAg, ^c^3 U/ml HBcrAg, ^d^3.1 U/ml HBcrAg

The presence of anti-HBs early in infection may block the detection of low levels of HBsAg by current assays since HBsAg assays rely on monoclonal antibodies directed at the S protein, particularly the immunodominant ‘a’ determinant, the same protein that is used in recombinant hepatitis B vaccines. We investigated whether using another region of the viral envelope (preS2) outside of the surface antigen targeted by vaccine antibodies would allow earlier detection of the virus. The research preS2 antigen assay allowed detection at day 65, earlier than current commercial HBsAg assays (Table 1).

The quantitative Lumipulse G HBcrAg assay detects various forms of the hepatitis B core protein, including HBeAg and the nucleocapsid core antigen, and has been shown to correlate with HBV DNA levels [14]. HBcrAg was first detected (≥ 3 log U/ml) in panel member 21, similar to the HBsAg assays (Fig. 1, Table 1). HBcrAg levels were low, increasing from 3 log U/ml at day 97 to 3.7 log U/ml in the last sample at day 115.

Panel 6272 was tested with a prototype ARCHITECT HBsAg assay having improved performance in analytical sensitivity and mutant and genotype detection without diminished specificity [11]. Among the serological assays evaluated, the prototype ARCHITECT HBsAg assay provided the earliest detection of panel 6272, becoming positive at day 51, 43–46 days earlier than current HBsAg assays (Fig. 1 and Table 1).

**Fig. 2:**
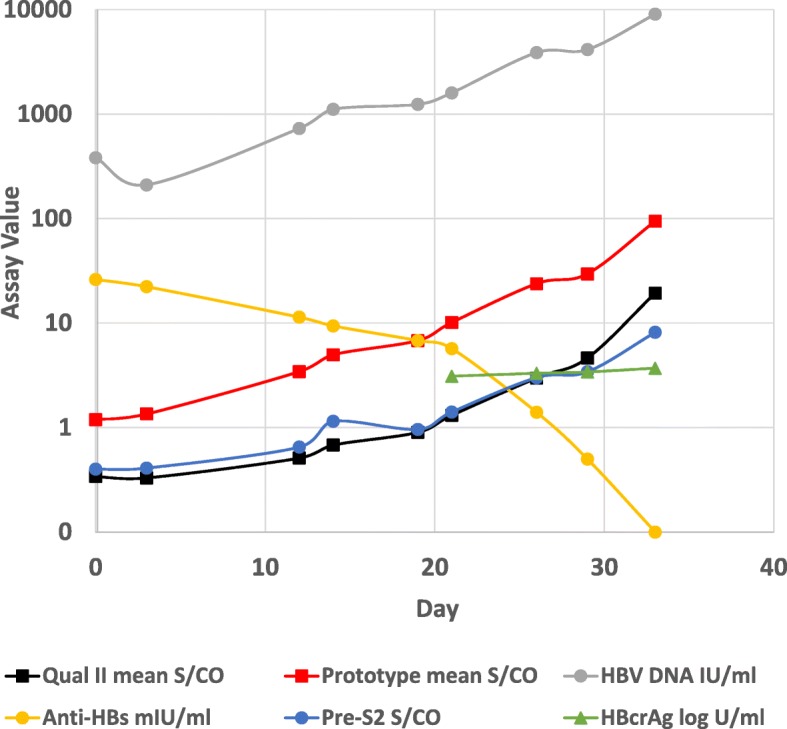
Serological and molecular profiles for panel 11,000. Panel 11,000 was tested with ARCHITECT HBsAg Qual II, a prototype ARCHITECT HBsAg assay, a research assay for preS2 antigen, ARCHITECT Anti-HBs, Lumipulse HBcrAg, and Abbott RealTime HBV. An S/CO value ≥ 1.0 was considered reactive for the HBsAg and preS2 assays. The limit of quantitation of the HBcrAg assay is 3 log U/ml; results for samples prior to day 21 were below the measuring range of the assay and were omitted from the figure. Anti-HBs levels ≥ 10 mIU/ml are considered protective

The molecular and serological profile of panel 11,000 (genotype A2, the same as the vaccine) is shown in Fig. 2. Like panel 6272, panel 11,000 had a low level of anti-HBs (26.1 mIU/ml) in the index donation but a higher HBV DNA concentration (383 IU/ml). During the 33 days of follow-up, HBV DNA levels increased slowly while anti-HBs levels steadily declined, dropping below 10 mIU/ml after day 20. Like panel 6272, panel 11,000 was negative for total anti-HBc, anti-HBc IgM, HBeAg, and anti-HBe at all time points. The preS2 antigen research assay detected HBsAg 7 days earlier than the commercial HBsAg assays while HBcrAg levels became detectable at a low level at the same time as the commercial HBsAg assays. As with panel 6272, the prototype ARCHITECT HBsAg assay provided the earliest serologic detection (at the first sample), at least 21 days earlier than current HBsAg assays (Table 1).

### Detection of HBsAg in the presence of anti-HBs

**Fig. 3:**
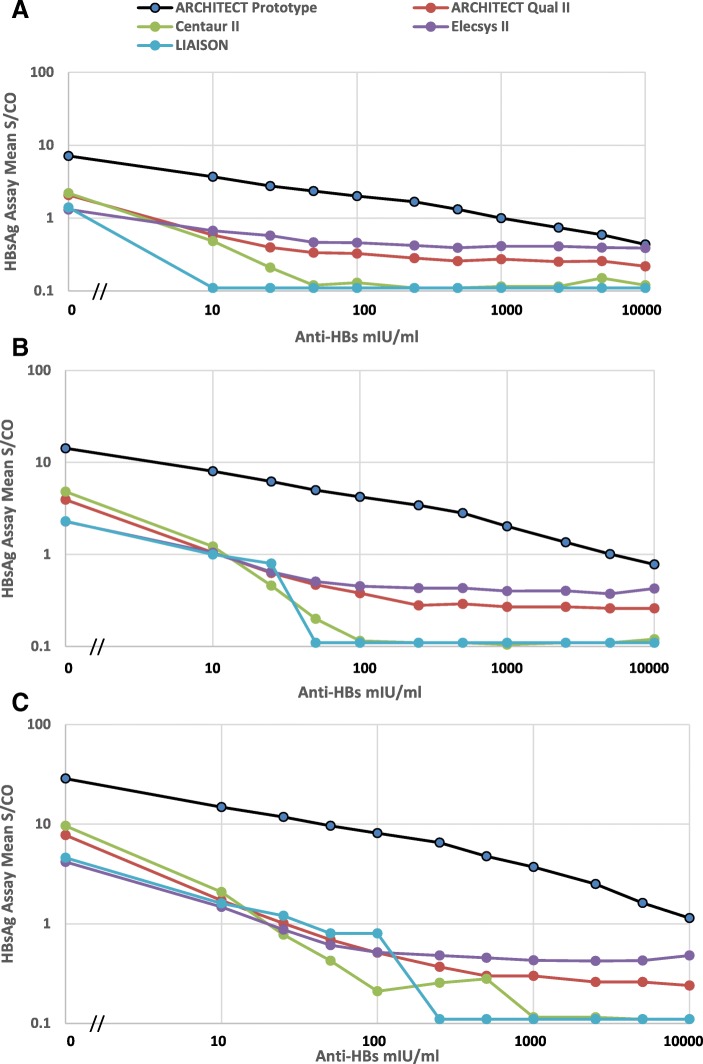
Detection of HBsAg in the presence of anti-HBs. Native HBsAg was mixed with antibody from a vaccinee to achieve HBsAg concentrations of 0.06 IU/ml (**a**), 0.11 IU/ml (**b**), and 0.23 IU/ml (**c**) with concomitant final anti-HBs concentrations ranging from 0 to 10,000 mIU/ml. Samples were then tested in duplicate with the ARCHITECT Qual II and prototype HBsAg assays, Centaur HBsAg II, Elecys HBsAg II, and LIAISON XL MUREX HBsAg Quant. Quantitative results for the LIAISON assay were converted to S/CO values. S/CO values ≥ 1.0 are considered reactive by the respective assays

In order to construct a panel of samples containing both HBsAg and anti-HBs, three levels of native HBsAg were combined with human vaccinee anti-HBs to give final concentrations of 0.06, 0.11, and 0.23 IU/ml HBsAg with anti-HBs ranging from 0 to 10,000 mIU/ml. The panel was tested in five HBsAg assays (Fig. 3 and Table 2). The levels of HBsAg chosen were low but above the analytical sensitivities of current HBsAg assays; the anti-HBs levels chosen spanned the range of those found in vaccine breakthrough infections and in many uninfected vaccinees. Anti-HBs concentrations of 10–25 mIU/ml, similar to the levels detected in panels 6272 and 11,000, rendered low levels of HBsAg undetectable in the ARCHITECT Qualitative II, Centaur II, Elecsys II, and LIAISON assays. In contrast, the ARCHITECT prototype assay required anti-HBs levels 250–400 times higher to block the detection of low level HBsAg.

**Table 2:** Detection of HBsAg in the Presence of Anti-HBs

HBsAg IU/ml	Anti-HBs level (mIU/ml) at which HBsAg became undetectable				
	ARCHITECT Prototype HBsAg	ARCHITECT HBsAg Qual II	Centaur HBsAg II	Elecsys HBsAg II	LIAISON XL MUREX HBsAg Quant.
0.06	2500	10	10	10	10
0.11	10,000	25	25	25	25
0.23	> 10,000	50	25	25	50

### Evaluation of mutations

All members of panels 6272 and 11,000 were sequenced in the preS1-preS2-S gene region. Results for 6272 revealed the presence of multiple substitutions in preS-S and in the reverse transcriptase (RT) region overlapping the envelope. PreS1 (aa 1–119) substitutions were: A54Q, L67F, I74V, T86A, S89P, T90A, I91V. PreS2 (aa 1–55) substitutions were: V32 L, N33S, A35V, N37 T, A47S, T49I, V53A, T54P. S (aa 1–226) substitutions were: D144DN, M198I, S207 N, V209 L. Substitutions in the overlapping RT region (aa 1–230) were: D7A, Y126H, M129 L, G152GE, V163I, I187L, V207 L, R217L. The D144N substitution within the immunodominant ‘a’ determinant occurred as a mixture with wild-type. The mutation was notable in that it represented 10–50% of codons in early samples (members 1–19) and then increased to ≥ 50% of codons coincidental with the marked decline in anti-HBs levels and the detection of HBsAg by current assays (Fig. 1). The corresponding reverse transcriptase substitution (rtG152E) is not within the active domain of the enzyme [5]. However, the rtV207L substitution is associated with antiviral drug resistance [15]. No mutations in preS1, preS2, S, or the overlapping RT region were found in any samples from panel 11,000. Sequencing of the precore and basal core promoter regions showed no mutations that might account for the HBeAg negativity observed for panels 6272 and 11,000.

## Discussion

The aim of this study was to characterize the molecular and serological profiles of two longitudinal vaccine breakthrough series and to evaluate alternative serological assays for the early detection of vaccine breakthrough. The course of vaccine breakthrough infections has been described in blood donors with a history of hepatitis B vaccination who were identified as HBV DNA positive in the absence of HBsAg and anti-HBc in samples taken near the onset of infection [3]. Viremia in these donors was transient and/or low level while HBsAg arose at apparently very low levels or remained negative. The two plasmapheresis donor series in the present study were positive for anti-HBs and HBV DNA and negative for HBsAg and anti-HBc on the index donation. Neither donor developed detectable anti-HBc, HBeAg, or anti-HBe during the follow-up periods (33 days and 115 days). HBeAg and anti-HBc usually appear 16–47 days and 30–52 days, respectively, after HBsAg is first detectable by current assays. Thus the follow-up period may have been insufficient to allow development of detectable HBeAg and anti-HBc, although in some vaccine breakthrough cases neither anti-HBc or HBsAg are detected even with prolonged follow-up [3]. Neither the precore stop codon mutation G1896A which abolishes HBeAg production or the basal core promoter mutations which cause reduced HBeAg production were found in any samples from either panel.

No HBsAg mutations were found in panel 11,000, a profile that has been reported for some vaccine breakthrough infections [4]. In contrast, panel 6272 displayed a number of substitutions in the preS-S region including a substitution at amino acid 144 (in the second loop of the ‘a’ determinant) where mutations are known to cause viral escape from vaccine-induced anti-HBs or hepatitis B immunoglobulin (HBIG). Mutations at this site are also associated with occult hepatitis B infection, escape from diagnostic assay detection, and impaired secretion of virions [16–19]. The most frequently reported mutations at amino acid 144 result in substitution of alanine, histidine, or glutamic acid for aspartic acid (D144A/E/H). The D144N mutation (aspartic acid to asparagine) is a recently identified, rare mutation that has been reported only four times previously: a vaccine breakthrough infection (genotype A2) in a patient who received a liver transplant from an anti-HBc positive donor, and in three HBV infected individuals from Iran, Tanzania, and China (genotypes D, A1, and C, respectively) [5, 20–22]. Only the first report was able to identify the clinical and diagnostic relevance of the mutation. To our knowledge, the current study is the first report of the D144N mutation in association with a vaccine breakthrough case in the United States. Hydrophilicity analysis of the second loop of the HBsAg major hydrophilic region by the Hopp & Woods method [23] indicated that a mutation to asparagine at amino acid 144 would result in a reduction in hydrophilicity similar to that for the A/H mutations, suggesting that the D144N mutation may affect the presentation or conformation of this epitope in the ‘a’ determinant.

We explored alternative immunoassays for detection of the vaccine breakthrough infections including HBcrAg (targeting the nucleocapsid) and a research preS2 antigen assay targeting the middle HBsAg protein which is not targeted by vaccine induced antibody. The research preS2 antigen assay allowed detection earlier than current HBsAg assays while the HBcrAg assay was equivalent to current HBsAg assays in time to detection. The most significant improvement in earlier detection was the prototype ARCHITECT HBsAg assay. This assay was previously shown to have improved analytical sensitivity, improved mutant and genotype detection, and specificity of 99.97–100% [11]. The prototype assay allowed detection of the index sample for panel 11,000 and detection 43 days earlier than current HBsAg assays for panel 6272. Rather than a lack of HBsAg expression during vaccine breakthrough infection as has been proposed in some reports, these results suggest that detectable HBsAg may be present and that the limiting factor is the HBsAg assay used in the testing.

Panels 6272 and 11,000 represent vaccine breakthrough in genotypes A1 and A2 respectively. Vaccine breakthrough cases from other HBV genotypes were not available for the present study. However, a previous study showed that the new assay has improved detection of HBsAg from genotypes A – H [11], suggesting that the assay may have improved detection of vaccine breakthrough cases regardless of genotype. Further studies are needed to confirm this.

It has been reported that HBsAg coexisting with anti-HBs occurs in 10–25% of chronic hepatitis B patients [24–26]. Some investigators have suggested that screening for vaccine induced anti-HBs in health care workers or in high-risk settings be accompanied by HBsAg testing since the presence of immune levels of anti-HBs does not exclude infection [26, 27]. The ability to detect HBsAg in these settings may be affected by the HBsAg assay used, requiring a test with broad mutant detection and sensitive detection of HBsAg. In the current study, testing of an HBsAg-anti-HBs panel showed that the new prototype HBsAg assay was able to detect low HBsAg levels (0.06–0.23 IU/ml) in the presence of anti-HBs concentrations 250–400 times higher (depending on the antigen concentration) than that tolerated by current HBsAg assays.

Further studies are in progress to evaluate the performance of the new prototype HBsAg assay in occult infections with and without the presence of anti-HBs. In addition, surveillance studies should continue to monitor vaccine breakthrough infections for the appearance of escape mutants to ensure the ability of diagnostic assays to detect them.

## Conclusions

We demonstrated that the new prototype ARCHITECT HBsAg assay is more sensitive in detecting vaccine breakthrough infections than current HBsAg assays or alternative immunoassay approaches such as testing for preS2 antigen and HBcrAg. We directly showed that the new assay, which is fully automated and requires no sample pre-treatment, has enhanced sensitivity in detecting HBsAg in the presence of anti-HBs. These findings have implications for improved diagnosis of HBV infection that may extend beyond vaccine breakthrough to include other HBV infections where HBsAg and anti-HBs may be present simultaneously as can occur in some occult and chronic infections.

## Abbreviations

anti-HBc IgM: IgM antibody to hepatitis B core antigen
anti-HBc: antibody to hepatitis B core antigen
anti-HBe: antibody to hepatitis B e antigen
anti-HBs: antibody to hepatitis B surface antigen
HBcrAg: Hepatitis B core-related antigen
HBeAg: Hepatitis B e antigen
HBsAg: Hepatitis B surface antigen
HBV DNA: Hepatitis B viral DNA
HBV: Hepatitis B virus
